# Willow (*Salix babylonica*) Extracts Can Act as Biostimulants for Enhancing Salinity Tolerance of Maize Grown in Soilless Culture

**DOI:** 10.3390/plants12040856

**Published:** 2023-02-14

**Authors:** Hande Mutlu-Durak, Yagmur Arikan, Bahar Yildiz Kutman

**Affiliations:** 1 Institute of Biotechnology, Gebze Technical University, Gebze 41400, Turkey; 2 Original Bio-Economy Resources Center of Excellence (OBEK), Gebze 41400, Turkey

**Keywords:** biostimulant, hydroponic, maize, salinity stress, salicylic acid, sustainability, willow extracts

## Abstract

Salinity negatively affects agricultural production by reducing crop growth and yield. Botanical biostimulants can be used as innovative and sustainable tools to cope with abiotic stress. In this study, salicylic acid (SA) (25 µM) and willow leaf (WL) (0.1 and 0.2%) and bark (WB) (0.1 and 0.2%) extracts were applied as plant-based biostimulants to hydroponically grown maize in the absence and presence of salinity stress (60 mM NaCl). The hormone-like activity and mineral composition of willow extracts were analyzed, and the effects of willow extracts on growth parameters, chlorophyll content, antioxidative enzyme activities, protein levels and mineral nutrient concentrations of maize plants were measured. Within the tested biostimulant applications, 0.2% WB, 0.1% WL and 0.2% WL gave the most promising results, considering the stress alleviating effects. The shoot biomass was increased up to 50% with 0.1% WL treatment and Na^+^ uptake was reduced with biostimulant applications under saline conditions. Under stress, the protein concentrations of maize leaves were enhanced by 50% and 80% with high doses of WB and WL applications, respectively. Results indicate that willow tree prunings can be valuable bio-economy resources, and aqueous extracts prepared from their leaves and barks can be used as effective and eco-friendly biostimulants.

## 1. Introduction

Globally, arable land is being rapidly lost or degraded because of shifts in land use due to urbanization and industrialization, climate change, salinization and acidification caused by wrong irrigation and fertilization practices, pollution and erosion [1,2]. Developing sustainable and eco-friendly crop production methods to feed the increasing population is a great challenge, which is closely associated with the sustainable development goals of the United Nations, including, but not limited to, zero hunger and poverty [3].

Salinity stress is one of the most important abiotic stress factors limiting agricultural production and threatening sustainability [4]. Due to salinity, crop production is affected at various growth stages including germination, vegetative growth and generative development [5,6]. Salinity stress has two major components: osmotic stress, followed by specific ion toxicities [7]. Under saline conditions, crop performance, mineral homeostasis and plant metabolism, such as protein synthesis and nitrogen assimilation, can be impaired [8]. Typically, salinity is associated with soil-based production systems where secondary salinization due to irrigation with poor quality water and excessive fertilization is a growing problem, but salinity stress is also a potential concern in soilless systems which are increasingly used for the competitive production of high-value crops in greenhouses as well as indoor farms [9].

One of the promising and novel applications to deal with abiotic stress conditions including salinity stress is the use of biostimulants in agricultural production [10]. Biostimulants, which contain a wide variety of bioactive compounds, have positive effects on plant metabolism such as in increasing the nutrient utilization efficiency and improving crop tolerance to biotic and abiotic stresses; although, their modes of action are not fully understood [11]. For this reason, biostimulant research is gaining popularity in the last decade and biostimulants are commonly used in agricultural production as an innovative and sustainable approach to cope with the global climate problem, reducing the use of harmful agrochemicals, increasing quality and yield, improving nutrient use efficiency and enhancing the stress tolerance of plants [12].

Biostimulants obtained from natural sources such as seaweed or plant extracts can be used in crop production as seed treatment agents [13], can be sprayed on the foliage [14,15] or applied to soils or soil substrates [16]. The biostimulant effects of various plant extracts obtained from roots [17], leaves [18,19], whole shoots [20] and barks [19,21] have been demonstrated in numerous studies. It has been shown that botanical biostimulants may have positive effects on germination, plant growth and development, nodule development, stress tolerance, fruit quality and yield of many different crop species [22], thanks to their bioactive substance content rich in amino acids, hormones, phenolic substances and carbohydrates [23]. Such extracts are potentially safe, natural and renewable inputs, whose effects can also be attributed to alterations in the phytohormone metabolism of target crops [24]. In a study investigating the effects of maize plants grown in a hydroponic system, extracts obtained from plant sources such as hawthorn leaves, red grape and blueberry fruits showed high biostimulant activities due to their bioactive components [25].

Willow (*Salix* sp.), which has been important for society due to its varied uses since the beginning of history and has been distributed almost all over the world, is a member of the *Salicaceae* family [26]. Especially, extracts of the bark and leaves of the willow tree have been used medicinally in the treatment of various illnesses as analgesics and anti-inflammatory agents [27]. Although the active ingredients of these extracts were not known at the beginning, it was later discovered that the extracts contained various salicylate compounds, including salicin, saligenin and salicylic acid (SA), as well as other secondary metabolites such as polyphenols, phenolic acid and terpenoids [28]. Salicin, the main active ingredient of willow bark (WB) and the original natural compound which eventually led to the development of the drug molecule acetylsalicylic acid commonly known as aspirin, is hydrolyzed by the enzyme beta-glucosidase to form d-glucose and saligenin [29]. Then, the oxidation of saligenin, which is also known as salicylic alcohol, will yield SA [30].

In addition to the medicinal properties mentioned above, it was recently shown that WB and willow leaf (WL) extracts obtained from weeping willow (*Salix babylonica*) can be used as seed treatment agents with biostimulant properties in maize [19]. The WB extracts can also be used as fungicides in agricultural applications due to their bioactive components including salicylic glycosides and salicylate [31]. Moreover, willow extracts may contain indole 3-butyric acid (IBA), the auxin hormone, and can therefore be useful in rooting applications [32]. The reported positive effects of willow extracts on root growth may also be related to the rich SA content [33].

Salicylic acid, as a phytohormone, plays a critical role in various physiological and biochemical processes in plants [34]. The application of SA as a plant growth regulator may enhance growth and development [35], synthesis of compatible solutes including proline and glycine betaine [36], chlorophyll content [37], flowering [38], photosynthesis [39], seed germination [40] and ion uptake and transportation [41]. Additionally, SA may act as a non-enzymatic antioxidant and enhance resistance to abiotic stress [42].

In studies on the relative salinity tolerance of various crops, maize (*Zea mays*) is typically referred to as moderately salt-sensitive crop with a threshold EC_e_ ranging from 1.3 to 1.8 dS m^−1^ and a yield reduction slope between 10.5 and 12.0%/dS m^−1^, according to the threshold and slope model initially proposed by Maas and Hoffman [43,44,45]. However, it is important to note that these values are all based on data from soil-based systems and ignore the potentially significant differences in the salinity tolerance of different cultivars. Salinity can cause a decrease in almost all growth parameters such as shoot and root length and biomass of maize [7]. Maize, which is together with wheat and rice one of the three major cereals feeding the world by providing up to 60% of the daily calories for human populations, is a critical crop for global food security as well as the economy [46,47]. According to the most recent FAO data available, the global maize production in 2020 exceeded 1.2 billion tons [48]. In addition to being a food crop, maize is also a critical feed and energy crop [49,50,51].

The aim of the present study is to document, for the first time and to the best of our knowledge, the biostimulant effects of aqueous WL and WB extracts, obtained from weeping willow, on maize in a model soilless system, and to compare their effects under control and saline conditions. Plants were grown hydroponically to enable the study of the effects of salinity and biostimulant applications on root growth. Because willow extracts are known to contain salicylate compounds including SA, the effects of willow extracts were compared to those of SA applications. The hormone-like activities of willow extracts and their effects on shoot and root growth parameters, leaf chlorophyll content, antioxidative enzyme activities, protein levels and macro- and micronutrient concentrations were determined to gain a comprehensive understanding of the physiological and nutritional aspects.

## 2. Results

**Figure 1:**
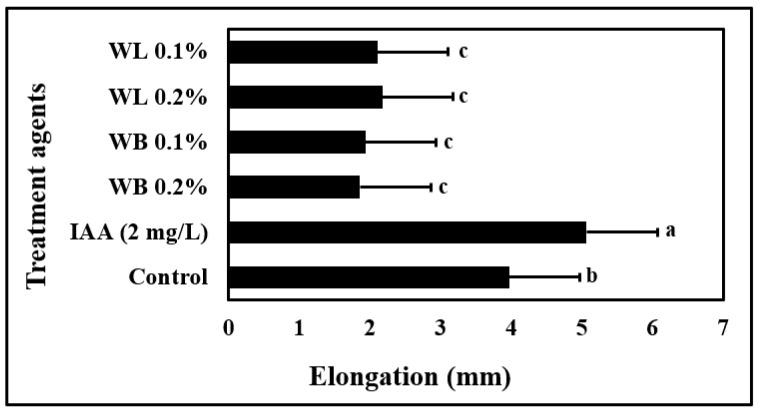
Maize coleoptile elongation rate in seven different solutions: WL, willow leaf extract (0.1% or 0.2% *w*/*v*); WB, willow bark extract (0.1% or 0.2% *w*/*v*); 2 mg/L of inodole-3-acetic acid (IAA); Control (Water). Different letters indicate significant differences according to Fisher’s protected LSD test (*p* = 0.05).

The effects of IAA and low (0.1%) and high (0.2%) concentrations of WL and WB extracts on maize coleoptile elongation rate were investigated to determine the auxin-like activity of willow extracts (Figure 1). When compared to control plants, a statistically significant increase in coleoptile elongation rate was observed only in plants treated with IAA. IAA application enhanced the maize coleoptile elongation rate by approximately 30% compared to control plants. Comparative analysis with IAA revealed that willow extracts did not show auxin-like activity.

**Figure 2:**
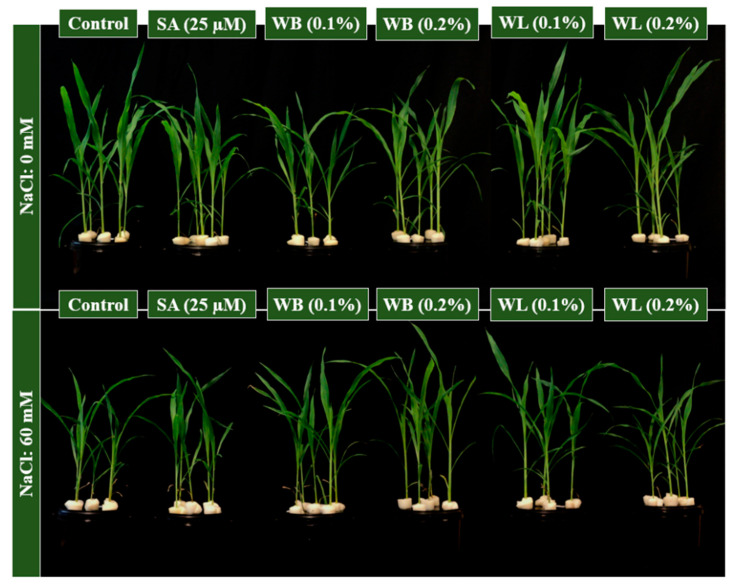
Effect of SA, salicylic acid (25 µM); WB, willow bark extract (0.1% or 0.2% *w/v*); WL, willow leaf extract (0.1% or 0.2% *w/v*) on plant height of 30 day-old hydroponically grown maize under control (0 mM) or salinity stress (60 mM) conditions.

**Figure 3:**
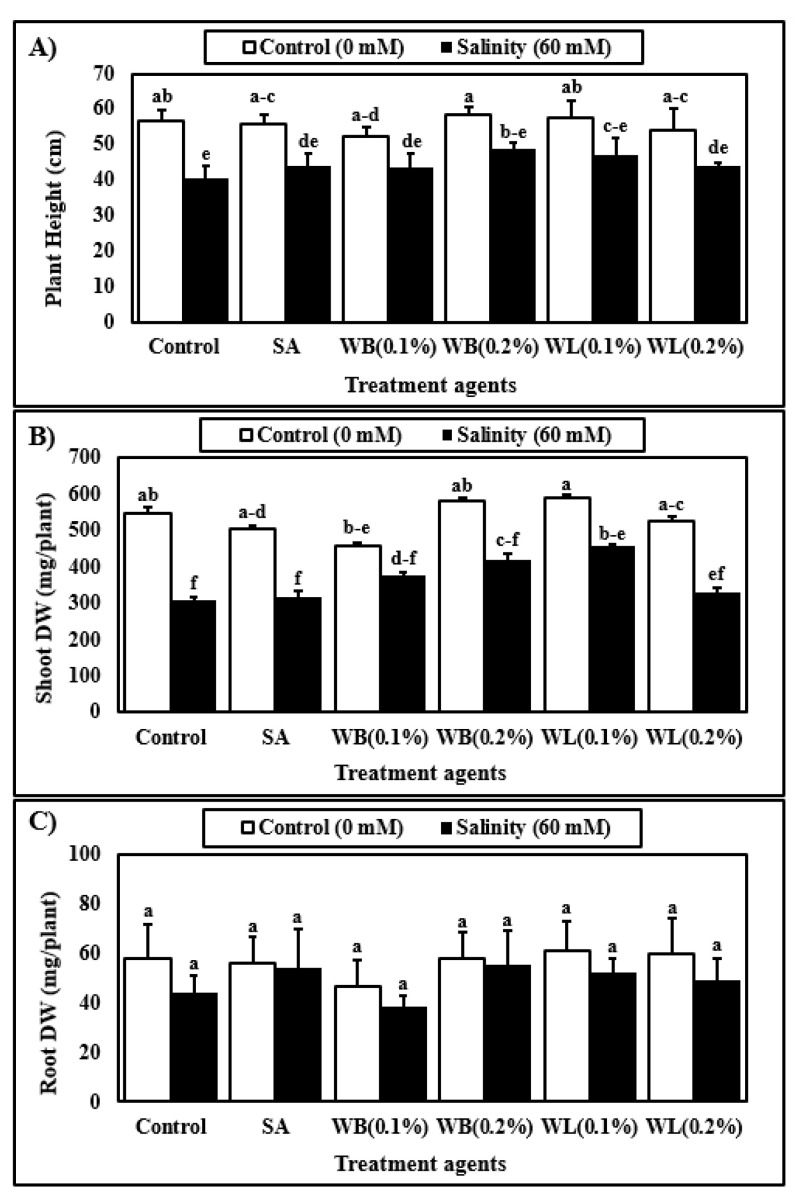
Effect of treatments with various agents (SA, salicylic acid (25 µM); WB, willow bark extract (0.1% or 0.2% *w/v*); WL, willow leaf extract (0.1% or 0.2% *w/v*)) on plant height (**A**), shoot dry weight (DW) (**B**), root dry weight (DW) (**C**) of 30-day-old maize (*Zea mays* cv. Caramelo) plants hydroponically grown in control (0 mM) or salinity stress (60 mM) conditions. Reported data are means of 4 independent biological replicates. Different letters indicate significant differences between means according to Tukey’s HSD test (p < 0.05). For two-way ANOVA results and the HSD scores used to separate the means, see Table 1.

Maize plants were grown under control and saline conditions and the effects of SA, WB and WL extracts were tested in deep water culture (Figure 2). Plants which were grown under saline conditions were smaller when compared to control plants (Figure 2 and Figure 3A,B). At the end of the experiment, among salinity-affected plants, those treated with the experimental biostimulants appeared generally taller (Figure 2).

According to two-way analysis of variance, biostimulant and salinity treatments affected plant height significantly (Table 1). On average, salinity reduced the plant height by approximately 20% (Figure 3A). Both salinity and biostimulant treatments significantly affected shoot DW of experimental plants according to ANOVA results, although the interaction was not significant (Table 1). On average, salinity reduced the shoot DW significantly by 30% (Figure 3B). When compared to the control plants, 0.1% WL applications enhanced the shoot DW by 22%. Under saline conditions, 0.1% WL application significantly enhanced the shoot DW by 48% when compared to non-treated plants. The low WL extract treatment also caused a significant increase in shoot DW when compared to SA treatment, but this increase was limited to 27% under saline conditions. The root DW was significantly affected by salinity stress (Table 1), which reduced the root DW of maize plants by 15% (Figure 3C). The biostimulant applications as well as the interaction of salinity and biostimulants did not cause any significant effect on root DW (Table 1 and Figure 3C).

The chlorophyll content was significantly affected by biostimulant and salinity applications, but their interaction did not have a significant effect on this trait (Table 1). Salinity stress increased the chlorophyl content of the maize plant by approximately 5% (Table 2). When averaged over salinity treatments to focus on just the significant main effect of the biostimulant applications, the chlorophyll content of the plants treated with 0.1% WB extract was reduced by 10% when compared to control plants.

Salinity, irrespective of the biostimulant treatments, significantly increased the specific SOD, APX and CAT activities by 12%, 18% and 20%, respectively, but it did not affect the specific GR activity (Table 1 and Table 3). The effects of biostimulant applications on all antioxidative enzyme activities were statistically significant according to ANOVA (Table 1). When averaged over salinity treatments, the specific GR activity was significantly reduced by SA as well as all experimental biostimulant applications by up to 60% (Table 1 and Table 3). In the case of specific SOD activity, the only significant change caused by the biostimulant applications with respect to control was a 29% reduction by 0.1% WB. The lowest specific APX and CAT activities were measured in plants treated with 0.2% WL application.

**Figure 4:**
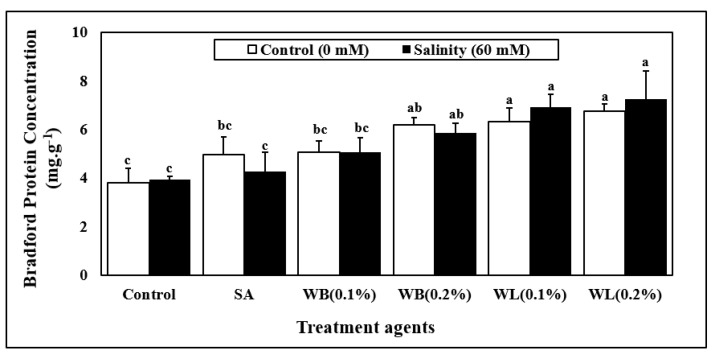
Changes in average protein concentration (590/450) (mg. g^−1^) of 30 day-old hydroponically grown maize (*Zea mays* cv. Caramelo) shoots in response to salinity (non-salinity stress, 0 mM; salinity stress, 60 mM) and biostimulant applications (SA, salicylic acid (25 µM); WB, willow bark extract (0.1% or 0.2% *w/v*); WL, willow leaf extract (0.1% or 0.2% *w/v*)). Reported data are means of 4 independent biological replicates. Different letters indicate significant differences between means according to Tukey’s HSD test (p < 0.05). For two-way ANOVA results and the HSD scores used to separate the means, see Table 1.

Leaf protein concentration was significantly affected by only biostimulant applications (Table 1, Figure 4). Irrespective of the salinity stress, the plants which did not receive any biostimulant treatment had the lowest protein levels. The SA treatment did not cause a significant increase in the protein levels. The high concentration of WB extract increased the leaf protein concentration by more than 50% on average, whereas the 0.1% WL and 0.2% WL applications resulted in even greater increases in the range of 70–80%.

According to ANOVA results, the shoot Na concentration as well as the Na/K ratio was significantly affected by the interaction of salinity and biostimulant treatments whereas the only significant effect on the shoot K concentration was the main effect of salinity (Table 1). Under saline conditions, the high concentration of WB and the low and high concentrations of WL extracts significantly reduced the Na concentration and Na/K ratios, but the SA treatment did not result in any significant changes in these parameters (Table 4). On average, salinity lowered the K concentration of experimental plants significantly by 15%.

The shoot macronutrient concentrations (P, Ca, Mg and S) of experimental plants are reported in Table 5. Irrespective of the biostimulant applications, salinity treatment caused an increase in shoot P and Mg concentrations and a decrease in shoot Ca and S concentrations (Table 1 and Table 5). Under saline conditions, the plants treated with a high dose of WL extract had significantly lower shoot P concentration when compared to control plants. When averaged over salinity treatments, 0.2% WL application significantly reduced Ca and enhanced S concentrations. Shoot Mg concentration was not affected by biostimulant treatments.

The micronutrient (Fe, Zn, Mn, Cu and Mo) concentrations of maize shoots are given in Table 6. In the absence of salinity stress, the shoot Fe and Zn concentrations were unaffected by the SA and experimental biostimulant applications (Table 6). However, salinity increased the shoot Fe and Zn concentrations of control (non-treated) plants by more than 50%, and under salinity, all willow extract applications except 0.1% WB resulted in significant decreases in Fe and Zn concentrations by up to 50%. The shoot Mn concentrations were elevated under salinity. The Cu concentration did not show a consistent response to biostimulant applications. Salinity stress caused, on average, an 80% reduction in shoot Mo concentration of maize plants. The shoot Mo concentrations were not affected by biostimulant applications.

## 3. Discussion

Plants must often deal with unfavorable environmental conditions which are known as abiotic or biotic stress factors. Salinity has an increasing importance among abiotic stress conditions due to secondary salinization associated with anthropogenic activities and climate change [4,5]. Biostimulant applications, which can enhance the stress tolerance of crop plants by stimulating plant metabolism in various ways, stand out among the numerous strategies used to cope with salinity stress due to their eco-friendly nature and immediate effectiveness [8,10,52]. Another advantage of biostimulants is that their potential benefits are not limited to stress conditions but can also be observed in the absence of any particular primary stressor [16,53,54].

To understand the mode of action of willow extracts, an auxin-like activity assay was performed by using the plant extracts (Figure 1). It was determined that willow extracts did not show an auxin-like activity. Instead, in response to willow extract applications, an inhibitory effect was observed. At the tips, browning was observed in coleoptiles treated with willow extracts (data not shown). This browning symptom might be due to the presence of rich phenolic substances in the willow extracts, which was reported in Mutlu-Durak and Yildiz Kutman [19]. Phenolic oxidation is known to be associated with browning [55,56]. As a phenolic compound, SA is also known to induce the production of other phenolic compounds [57], which may also contribute to this outcome. It is also important to note that SA and IAA compete for the same metabolites in the plant’s metabolism, and therefore plant tissues which are high in SA compounds may not be rich in IAA [30,58].

Maize is known to be susceptible to salinity stress, and Na ions are reported to inhibit the growth and development of the maize plant grown in the hydroponic system [7]. In this study, the stimulatory effects of 0.1% WL on the growth of the maize plant were observed both under control and stress conditions (Figure 2 and Figure 3). As shown in Figure 2, plants treated with willow extracts are more resistant to salinity stress when compared to control and SA-treated plants. Studies on many different plants have shown that depending on the concentration and treatment method, SA and its derivatives, which are known as salicylates, have either stimulating or inhibiting effects on plant growth and development under control and stress conditions [30,59]. These compounds are known to play an important role in primary and secondary metabolism by triggering metabolic and physiological activities in plants [60]. Many studies have reported that SA is an effective phytohormone in increasing root length and growth [61]. It is also reported that aspirin, a close relative of SA, improves rooting in bean plants [62]. In this study, however, WB and WL extracts did not exert a significant effect on root DW (Figure 3C). Since auxin activity is known to enhance root growth, the lack of auxin-like activity in the willow extracts may be one of the reasons behind this finding (Figure 1).

It was observed that salinity stress inhibited shoot and root growth of the maize plant in a soilless culture (Figure 3). Consistent with our results, a study by Bose et al. documented that the salinity level of 8 dS m^−1^ (equivalent to 80 mM) reduced the shoot and root growth of maize in solution culture [5]. Based on the findings of this study, willow extracts were effective in alleviating the negative effects of salinity stress, but the positive effects were concentration-dependent (Figure 3). Among others, the low (0.1%) dose application of WL extract was shown to be the most effective treatment for improving the vegetative growth of maize (Figure 2 and Figure 3). Applications of biostimulants of different kinds were reported to mitigate the adverse effects of salinity in maize [10,52].

Under stress conditions such as salinity, in response to the oxidative stress conditions, an increase in reactive oxygen species (ROS) levels can be observed [63]. If the ROS levels are not controlled properly, DNA, membrane, chlorophyll and protein damage can occur in the plant system [64]. Plants can cope with high ROS levels with the help of antioxidative enzymes such as SOD, CAT, GR and APX [65]. According to Table 1 and Table 3, the specific activities of all enzymes except GR were increased by salinity, indicating that salinity imposed an oxidative stress on the plants which, in response, had to upregulate their enzymatic ROS-scavenging system. On the other hand, the specific activities of all antioxidative enzymes of interest were significantly reduced by all or some specific treatments, which can be interpreted as a reduced oxidative burden and therefore a reduced need for enzymatic ROS-scavenging activity [66,67]. Exogenous SA applications may cause positive or negative effects on the activities of anti-oxidative enzymes depending on the conditions [61]. Here, only the GR activity was affected by the SA treatment (Table 3). So, at least at the level tested in this study, the effects of the willow extracts on the antioxidative defense machinery could not be fully attributed to their SA content.

In this study, the stimulating effects of willow extracts on leaf protein levels of maize were also observed (Figure 4). Increased leaf protein concentration may suggest that the application of willow extracts to the nutrient solution may enhance yet-to-be-determined aspects of nitrogen and protein homeostasis, possibly including nitrate uptake, assimilation, as well as protein synthesis. In another study, a plant-based biostimulant obtained from alfalfa applied to maize plants grown in a hydroponic system was reported to have positive effects on nitrogen metabolism [52]. Moreover, SA was shown to have positive effects on nitrogen metabolism and to enhance protein concentrations of plants when it is used at optimized rates [68,69].

Part of the ionic stress associated with salinity is the direct toxicity of ions, whereas, another part is the indirect effects of the potentially toxic ions on the essential nutrients due to competition and/or non-functional replacement. For cereals in general and for maize in particular, Na and not Cl is considered the primarily toxic ion under salinity stress [7,70]. The Na/K ratio in shoot tissues is a key quantitative indicator of salinity tolerance in plants [71,72,73]. One of the most important mechanisms of salinity tolerance in glycophytes is the efficient exclusion of Na [71]. So, glycophytes with relatively lower Na/K ratios are considered to be more tolerant to salinity stress [74]. As shown in Table 4, plants which were grown with willow extract applications had reduced Na concentrations in their shoot tissues. Plants treated with willow leaf extracts had the lowest Na/K ratios compared to other treatments. So, the stress-alleviating effect of willow extracts can at least partly be attributed to the decrease in Na accumulation and the maintenance of a relatively lower Na/K ratio. Sodium can also compete with Ca and Mg by affecting uptake, translocation and physiological utilization of these essential elements and cause nutritional problems [7,70]. Application of willow extracts did not significantly affect the concentrations of Ca and Mg (Table 5).

In wheat and maize under salinity stress, SA applications were reported to significantly decrease the Na concentration [75,76]. Here, however, a significant effect of SA application on shoot Na concentrations could not be detected, possibly due to the tested concentration.

Although the willow extracts contained trace amounts of macronutrients, they were negligible when the extracts were applied at relatively low amounts (Table 7). Willow extracts are poor in macro- and micronutrients. In the absence of any biostimulant treatment, irrespective of salinity, the concentrations of all measured macro- and micronutrients in maize shoots were in ranges considered adequate [77]. Based on these results, the positive effects obtained in response to willow extract applications could not be explained by the correction of any nutrient deficiency. This also supports that the willow extract applications work as biostimulants rather than fertilizers, and contribute to the induction of different metabolic pathways beyond providing nutrients to the plant.

## 4. Materials and Methods

### 4.1. Plant material and Growth Conditions

Maize, which is one of the most important staple crops and it is known to be moderately sensitive to salinity, was selected as a model crop for this project. In this study, the maize seeds (*Zea mays* cv. Caramelo F1) obtained from May Seed, Bursa, Turkiye, were used as plant material. The germination percentage of the Caramelo species, which are suitable for both fresh consumption and industrial use, was determined as 90%. This species is known to be an early, dwarf and hybrid sweet maize.

The study was carried out in a growth chamber under controlled conditions (25 °C/light for 16 h and 20 °C/dark for 8 h and relative humidity (light/dark): 60/70).

### 4.2. Preparation of Willow Tree Extracts

The willow extracts were prepared according to the method previously described in the article [19]. For extract preparation, fresh WL and WB samples were obtained in the fall of 2018 from the pruned branches of a grown weeping willow tree (*Salix babylonica*) in the Tuzla area, of which 100 g of WL or WB were sliced into small pieces, and the total volume was brought to 1 L using dH_2_O to produce 10% willow extracts for leaf and bark samples. For extraction, the mixture was kept at 90 °C for 30 min while being agitated continuously at 400 rpm. Using a cheesecloth, the solution was filtered; the filtrates were then centrifuged at 4000 rpm. After the aqueous extraction was completed, the extracts were stored at −20 °C until further usage. The total phenolics, salicin, saligenin and SA concentrations of the extracts were reported in Mutlu-Durak and Kutman [19]. The mineral composition of the extracts is given below.

### 4.3. Mineral Element Characterization of Willow Extracts

The mineral elements of willow extracts were measured as explained in the element analysis section, without the wet-digestion step by using ICP-OES, and listed in Table 7.

### 4.4. Auxin-like Activity of Willow Extracts

To determine the hormonal effects of willow extracts, “corn coleoptile elongation rate test” was used, as described by Colla et al. [54]. In this method, maize seeds were germinated in the dark for 1 week under growth chamber conditions. The corn coleoptiles were grown until they were 2-3 cm long. The apical parts (3–4 mm) of the coleoptiles were removed, and from every coleoptile, 2 cm standard pieces were taken for the test. The collected coleoptile fragments were placed in petri dishes with a diameter of 10 cm containing 20 mL of solutions. The solutions that were used for this test contained indole-3-acetic acid (IAA), purified water or the willow extracts. For each application, there were 4 independent replicates each containing 5 coleoptile fragments and the trial was carried out for 48 h. At the end of this period, the coleoptile lengths were measured using the Image J program and auxin-like effects of the willow extracts were calculated in comparison to the IAA application.

### 4.5. Solution Culture Experiment

For the solution culture experiment, maize seeds were germinated in perlite medium, which was moistened with 1 mM Ca(NO_3_)_2_ for 7 d in a growth chamber before being transferred to the nutrient solution. After germination, plants were transferred to hydroponic culture pots (5 plants per pot) which were filled with 4.5 L of half-strength nutrient solution and continuously aerated. After 2 days, the solution culture was refreshed with full-strength nutrient solution. The full strength nutrient solution contained 1.2 mM K_2_SO_4_, 4 mM Ca(NO_3_)_2_.4H_2_O, 0.2 mM KH_2_PO_4_, 0.75 mM MgSO_4_.7H_2_O, 0.1 mM KCl, 100 μM Fe (in the form of FeEDTA), 2 μM H_3_BO_3_, 1 μM MnSO4.H_2_O, 1 μM ZnSO_4_.7H_2_O, 0.6 μM CuSO_4_.5H_2_O, 0.50 μM (NH_4_)_6_Mo_7_O_24_.4H_2_O. To prevent Fe deficiency, which is a common problem for maize grown under soilless culture conditions, FeSO_4_ (10 µM) was added to the nutrient solution at an interval of 3 days. Every 5 days after the solution was changed, the hydroponic culture was refreshed with full-strength nutrients. For salinity treatment, 60 mM NaCl was mixed with the nutrient solutions. At the same time, SA (2.5 μM), low (0.1%) and high (0.2%) levels of WB and WL extracts were added to the nutrient solutions of experimental pots. The experiment had a completely randomized design with four replicates for each treatment group. The experimental plants were grown for 30 days after sowing (DAS).

At the end of the experimental period (30 DAS), plant shoot heights were measured for all experimental plants. After harvest, the shoots of three plants were pooled and stored at −80 °C until they were used for Bradford protein and antioxidative enzyme activities analysis. The shoots of the remaining two plants and roots were washed with distilled water. Both the root and shoot samples were dried in an oven for 3 days at 60 ºC. The dry weights of shoot and root samples were measured. Dry shoot samples were used for the mineral element analysis.

### 4.6. Chlorophyll Content Analysis

Chlorophyll contents in maize leaves were non-destructively measured from fully expanded leaves by using a portable device DUALEX SCIENTIFIC+TM (FORCE-A, Orsay, France) before the harvest.

### 4.7. Element Analysis

For element analysis, dried shoot samples were ground and approximately 0.2 g of plant samples were placed in digestion tubes. For acid digestion, 2.0 mL of 30% hydrogen peroxide (H_2_O_2_) and 5.0 mL of 65% nitric acid (HNO_3_) were applied to samples. A microwave device (MarsExpress; CEM Corp., Matthews, NC, USA) was used for wet digestion of plant samples. Once the samples were thoroughly cooled, the total sample volume was adjusted to 20 mL by adding double-deionized water and it was filtered by filter papers (Macherey-Nagel, Ø125 mm, blue band). The concentrations of macro and micronutrients as well as Na of plant samples were measured by using inductively coupled plasma optical emission spectrometry (ICP-OES) (Agilent 5800, Vista-Pro Axial, Varian, Australia). Certified standard reference materials received from the National Institute of Standards and Technology (Gaithersburg, MD, USA) were used for the accuracy of element analyses.

### 4.8. Extraction for Protein and Antioxidative Enzyme Assays

Potassium phosphate (K-P) buffer with a pH of 7.6 was prepared by combining 50 mM KH_2_PO_4_ and 50 mM K_2_HPO_4_. 0.1 mM EDTA Titriplex-III was added to the solution to create the extraction buffer, which was then chilled. In 5 mL of 50 mM K-P buffer, 0.5 g of maize leaf samples were homogenized. The homogenates were centrifuged at 4600 min^−1^ for 15 min at 4 °C, and the supernatants were transferred to microcentrifuge tubes and centrifuged at 15.000 min^−1^ for 15 min at 4 °C. These supernatants were utilized to measure the amounts of proteins and antioxidant enzymes (SOD, GR, AP, and CAT).

### 4.9. Determination of Antioxidative Enzyme Activities

The superoxide dismutase (SOD) activity was determined spectrophotometrically by using the method described by Giannopolitis and Ries [78,79]. In this procedure, in a glass tube, 0.5 mL of Na_2_CO_3_, L-methionine, NBT, 0.05 mL of crude sample extract (1:10 diluted) and 0.5 mL riboflavin were mixed with 2.95 mL of K-P buffer. During preparation, all the chemicals were kept in the dark due to light sensitivity. Glass tubes were placed in the growth chambers once the riboflavin was added, and they were kept under a light source for 8 min. A spectrophotometer (Cary 300 Bio, Varian, Australia) set at 560 nm was used for measurements.

The glutathione reductase (GR) activity in shoot samples was measured by using the assay described by Carlberg and Mannervik [79,80]. In total, 0.7 mL of K-P buffer, 0.1 mL of Oxidized Glutathion (GSSG), 0.1 mL of 0.45 mM H_2_O_2_, 0.1 mL of diluted crude sample extract (1:10), and 0.1 mL of NaDPH-Na_4_ were mixed and the mixture was used to measure the activity of the GR enzyme. The GR activity was calculated by monitoring for 2 min at 340 nm and the average depletion rate of NADPH-Na_4_ was calculated accordingly.

Ascorbic acid (C_6_H_8_O_6_) was added to 0.7 mL of K-P buffer, 0.1 mL of 12 mM H_2_O_2_ and 0.1 mL of the crude sample extract (1:40 diluted) to assess ascorbate peroxidase (APX) activity, according to the analysis by the method of Nakano and Asada [81,82]. Spectrophotometric measurements of absorbance values at 290 nm were used to calculate the average depletion rate of L-ascorbic acid.

Catalase (CAT) activity was measured in plant shoot samples by using a slightly modified version of the spectrophotometric assay reported by Chance and Maehly [83,84]. For this purpose, 0.1 mL of 100 mM H_2_O_2_, 0.1 mL of crude sample extract and 0.8 mL of K-P buffer were combined (1:40 diluted). To determine the average rate of H_2_O_2_ breakdown, the absorbance of this mixture was monitored for 2 min at 240 nm.

### 4.10. Total Bradford Protein Analysis

The Bradford technique was used to calculate the total protein concentration and bovine serum albumin was used as the reference [85]. To prepare Bradford reagent, 0.1 g Coomassie Brilliant Blue G-250 was dissolved in 50 mL ethanol and was mixed with 100 mL 85% ortho-phosphoric acid. The reagent was kept at 4 °C for 24 h and then used for the assay. Protein standards (0, 100, 200, 400 and 800 ppm) were prepared by dissolving bovine serum albumin in K-P buffer, which is described above. Then, 5 mL of reagent was added to 0.1 mL sample or standard and vortexed. After 10 min, the multiwavelength absorbance was read at 450, 590 and 595 nm.

### 4.11. Statistical Analysis

The JMP software (version 14.0.0) was utilized for the statistical analysis. Analysis of variance was used to determine the significance of the treatments’ effects and their interactions on the reported attributes for each experiment (ANOVA). Means were separated by Fisher’s protected least significant difference (LSD) test at 5% significance in single-factor experiments, whereas Tukey’s honestly significant difference (HSD) test at 5% significance was used for this purpose in multi-factor settings.

## 5. Conclusions

From the promising results obtained in this study, it can be concluded that WB and WL extracts can be used as biostimulants to ameliorate the negative effects of salinity stress in maize. Among the tested willow bark and leaf applications, 0.2% WB, 0.1% WL and 0.2% WL gave the best results considering the growth promoting and/or stress alleviating effects. The beneficial effects observed with willow extract applications cannot be explained by just their SA content because the same benefits could not be obtained with the exogenous application of pure SA. Willow extracts were able to reduce Na uptake and thus enhance the K/Na ratio of maize shoots. Applications of willow extracts also enhanced the protein concentrations of plant shoots, suggesting a possible role of willow extracts in N metabolism, which deserves further investigation. Our results indicate that willow tree prunings can be valuable bio-economy resources and aqueous extracts prepared from their leaves and barks can be used as effective and eco-friendly biostimulants.

## Figures and Tables

**Table 1:** Significance of the effects of biostimulants (A), salinity (B) and their interactions on reported traits of hydroponically grown maize (*Zea mays* cv. Caramelo) plants (30 days after sowing (DAS)) according to two-way analysis of variance (ANOVA) (α = 0.05).

Source of Variation	DF	Plant Height		Shoot DW		Root DW	
		F Pr.	HSD_0.05_	F Pr.	HSD_0.05_	F Pr.	HSD_0.05_
Biostimulant (A)	5	0.019	5.4	<0.001	76.9	0.123	17.0
Salinity (B)	1	<0.001	2.1	<0.001	29.9	0.024	6.6
A × B	5	0.385	8.9	0.055	126.2	0.885	27.9
Source of Variation	DF	Chlorophyll Content		SOD		GR	
		F Pr.	HSD_0.05_	F Pr.	HSD_0.05_	F Pr.	HSD_0.05_
Biostimulant (A)	5	0.013	3.0	<0.001	5.6	<0.001	0.041
Salinity (B)	1	0.010	1.2	0.010	2.2	0.522	0.016
A × B	5	0.496	4.9	<0.001	9.2	0.188	0.068
Source of Variation	DF	APX		CAT		Protein	
		F Pr.	HSD_0.05_	F Pr.	HSD_0.05_	F Pr.	HSD_0.05_
Biostimulant (A)	5	<0.001	0.84	<0.001	20.5	<0.001	0.91
Salinity (B)	1	0.009	0.33	<0.001	8.0	0.861	0.35
A × B	5	0.685	1.38	0.144	33.7	0.278	1.49
Source of Variation	DF	Na		K		Na/K	
		F Pr.	HSD_0.05_	F Pr.	HSD_0.05_	F Pr.	HSD_0.05_
Biostimulant (A)	5	<0.001	0.042	0.249	0.93	<0.001	0.008
Salinity (B)	1	<0.001	0.016	<0.001	0.36	<0.001	0.003
A × B	5	<0.001	0.068	0.469	1.52	<0.001	0.013
Source of Variation	DF	P		Ca		Mg	
		F Pr.	HSD_0.05_	F Pr.	HSD_0.05_	F Pr.	HSD_0.05_
Biostimulant (A)	5	0.088	0.258	<0.001	0.125	0.297	0.068
Salinity (B)	1	<0.001	0.100	<0.001	0.049	0.004	0.026
A × B	5	0.010	0.422	0.521	0.205	0.863	0.111
Source of Variation	DF	S		Fe		Zn	
		F Pr.	HSD_0.05_	F Pr.	HSD_0.05_	F Pr.	HSD_0.05_
Biostimulant (A)	5	0.008	0.080	<0.001	22.4	<0.001	11.8
Salinity (B)	1	<0.001	0.031	<0.001	8.7	<0.001	4.6
A × B	5	0.069	0.132	0.017	36.7	<0.001	19.4
Source of Variation	DF	Mn		Cu		Mo	
		F Pr.	HSD_0.05_	F Pr.	HSD_0.05_	F Pr.	HSD_0.05_
Biostimulant (A)	5	0.027	31.7	0.005	1.8	0.442	0.301
Salinity (B)	1	<0.001	12.3	0.907	0.7	<0.001	0.117
A × B	5	0.971	51.9	0.397	3.0	0.264	0.493

**Table 2:** Effect of treatments with various agents (SA, salicylic acid (25 µM); WB, willow bark extract (0.1% or 0.2% *w/v*); WL, willow leaf extract (0.1% or 0.2% *w/v*)) on chlorophyll content of 30-day-old maize (*Zea mays* cv. Caramelo) plants hydroponically grown in control (0 mM) or salinity stress (60 mM) conditions.

Treatment	Salinity (mM)	Chlorophyll Content (µg/cm^2^) 30 DAS
Control	0	32.9 ± 0.6 ab
	60	33.2 ± 2.6 a
SA (25 µM)	0	32.3 ± 0.6 ab
	60	33.4 ± 2.9 a
WB (0.1%)	0	28.1 ± 1.1 b
	60	31.9 ± 0.8 ab
WB (0.2%)	0	30.4 ± 1.6 ab
	60	30.8 ± 3.4 ab
WL (0.1%)	0	29.9 ± 1.9 ab
	60	32.0 ± 1.5 ab
WL (0.2%)	0	29.8 ± 2.7 ab
	60	31.4 ± 0.9 ab

Reported data are means of 4 independent biological replicates. Different letters indicate significant differences between means according to Tukey’s HSD test (*p* < 0.05). For two-way ANOVA results and the HSD scores used to separate the means, see Table 1.

**Table 3:** Changes in specific activity of antioxidative enzymes (SOD, GR, APX, CAT) of 30 day-old hydroponically grown maize (*Zea mays* cv. Caramelo) shoots in response to salinity (non-salinity stress, 0 mM; salinity stress, 60 mM) and biostimulant applications (SA, salicylic acid (25 µM); WB, willow bark extract (0.1% or 0.2% *w*/*v*); WL, willow leaf extract (0.1% or 0.2% *w/v*)).

Treatments	Salinity (mM)	SOD	GR	APX	CAT
		(U g^−1^ FW)	(-µmol [NADPH] g^−1^ FW min^−1^)	(-µmol H_2_O_2_ g^−1^ FW min^−1^)	
Control	0	27.2 ± 6.0 a–d	0.182 ± 0.074 d	2.46 ± 0.12 ab	104 ± 18 b–d
	60	27.8 ± 2.0 a–d	0.159 ± 0.012 cd	3.04 ± 0.74 bc	106 ± 9 b–d
SA (25 µM)	0	20.6 ± 2.5 b–e	0.079 ± 0.010 ab	2.59 ± 0.94 a–c	102 ± 18 b–d
	60	27.4 ± 2.6 a–d	0.114 ± 0.031 a–c	2.99 ± 0.51 a–c	128 ± 18 d
WB (0.1%)	0	19.2 ± 3.3 de	0.126 ± 0.029 a–d	2.95 ± 0.49 a–c	82 ± 30 ab
	60	20.0 ± 2.7 c–e	0.099 ± 0.015 a–c	3.92 ± 1.04 c	119 ± 26 cd
WB (0.2%)	0	15.6 ± 2.0 e	0.115 ± 0.030 a–d	2.59 ± 0.78 a–c	89 ± 3 a–c
	60	28.7 ± 9.2 a–c	0.130 ± 0.051 b–d	3.02 ± 0.41 a–c	106 ± 7 b–d
WL (0.1%)	0	34.8 ± 2.1 a	0.093 ± 0.024 a–c	2.44 ± 0.23 ab	95 ± 16 a–d
	60	30.6 ± 4.0 a	0.074 ± 0.023 ab	2.49 ± 0.49 ab	103 ±5 b–d
WL (0.2%)	0	29.8 ± 2.1 ab	0.072 ± 0.012 ab	1.65 ± 0.41 a	62 ± 9 a
	60	30.2 ± 3.4 a	0.061 ± 0.013 a	1.91 ± 0.30 ab	74 ± 10 ab

Reported data are means of 4 independent biological replicates. Different letters indicate significant differences between means according to Tukey’s HSD test (*p* < 0.05). For two-way ANOVA results and the HSD scores used to separate the means, see Table 1.

**Table 4:** Changes in shoot Na, K concentrations and Na/K ratio of 30 day-old hydroponically grown maize (*Zea mays* cv. Caramelo) plants in response to salinity (non-salinity stress, 0 mM; salinity stress, 60 mM) and biostimulant applications (SA, salicylic acid (25 µM); WB, willow bark extract (0.1% or 0.2% *w/v*); WL, willow leaf extract (0.1% or 0.2% *w/v*)).

Treatments	Salinity (mM)	Na (%)	K (%)	Na/K
Control	0	0.0002 ± 0.0001 d	6.95 ± 0.11 a–c	0.0000 ± 0.0000 c
	60	0.2999 ± 0.0455 a	5.57 ± 0.66 c	0.0541 ± 0.0082 a
SA (25 µM)	0	0.0004 ± 0.0001 d	7.08 ± 0.37 a–c	0.0001 ± 0.0000 c
	60	0.2599 ± 0.0323 ab	6.54 ± 1.27 a–c	0.0407 ± 0.0090 ab
WB (0.1%)	0	0.0009 ± 0.0003 d	7.25 ± 0.28 a	0.0001 ± 0.0000 c
	60	0.2334 ± 0.0360 ab	5.61 ± 0.11 c	0.0417 ± 0.0068 ab
WB (0.2%)	0	0.0007 ± 0.0001 d	7.17 ± 0.21 ab	0.0001 ± 0.0000 c
	60	0.2153 ± 0.0321 bc	5.94 ± 0.32 a–c	0.0364 ± 0.0065 b
WL (0.1%)	0	0.0016 ± 0.0004 d	7.16 ± 0.23 a–c	0.0002 ± 0.0001 c
	60	0.1944 ± 0.0506 bc	6.47 ± 1.29 a–c	0.0303 ± 0.0077 b
WL (0.2%)	0	0.0008 ± 0.0001 d	6.76 ± 0.45 a–c	0.0001 ± 0.0000 c
	60	0.1598 ± 0.0338 c	5.72 ± 0.44 bc	0.0284 ± 0.0078 b

Reported data are means of 4 independent biological replicates. Different letters indicate significant differences between means according to Tukey’s HSD test (*p* < 0.05). For two-way ANOVA results and the HSD scores used to separate the means, see Table 1.

**Table 5:** Changes in shoot macronutrient (P, Ca, Mg, S) concentrations of 30 DAS hydroponically grown maize (*Zea mays* cv. Caramelo) plants in response to salinity (non-salinity stress, 0 mM; salinity stress, 60 mM) and biostimulant applications (SA, salicylic acid (25 µM); WB, willow bark extract (0.1% or 0.2% *w/v*); WL, willow leaf extract (0.1% or 0.2% *w/v*)).

Treatments	Salinity (mM)	P (%)	Ca (%)	Mg (%)	S (%)
Control	0	1.08 ± 0.05 c	0.80 ± 0.02 a	0.35 ± 0.01 a	0.60 ± 0.01 bc
	60	1.85 ± 0.21 a	0.65 ± 0.12 ab	0.39 ± 0.06 a	0.52 ± 0.07 c
SA (25 µM)	0	1.07 ± 0.06 c	0.71 ± 0.03 ab	0.34 ± 0.01 a	0.61 ± 0.02 bc
	60	1.76 ± 0.29 ab	0.66 ± 0.15 ab	0.42 ± 0.09 a	0.59 ± 0.11 bc
WB (0.1%)	0	1.22 ± 0.18 c	0.80 ± 0.08 a	0.37 ± 0.03 a	0.63 ± 0.02 a–c
	60	1.76 ± 0.27 ab	0.61 ± 0.06 ab	0.40 ± 0.04 a	0.52 ± 0.03 c
WB (0.2%)	0	1.22 ± 0.05 c	0.74 ± 0.09 ab	0.36 ± 0.02 a	0.65 ± 0.02 a–c
	60	1.46 ± 0.09 a–c	0.55 ± 0.06 bc	0.39 ± 0.03 a	0.57 ± 0.03 bc
WL (0.1%)	0	1.18 ± 0.07 c	0.72 ± 0.04 ab	0.37 ± 0.01 a	0.67 ± 0.03 ab
	60	1.78 ± 0.28 ab	0.56 ± 0.13 bc	0.40 ± 0.09 a	0.57 ± 0.11 bc
WL (0.2%)	0	1.15 ± 0.06 c	0.59 ± 0.05 bc	0.32 ± 0.02 a	0.76 ± 0.05 a
	60	1.38 ± 0.13 bc	0.40 ± 0.06 c	0.35 ± 0.03 a	0.56 ± 0.01 bc

Reported data are means of 4 independent biological replicates. Different letters indicate significant differences between means according to Tukey’s HSD test (*p* < 0.05). For two-way ANOVA results and the HSD scores used to separate the means, see Table 1.

**Table 6:** Changes in micronutrient (Fe, Zn, Mn, Cu, Mo) concentrations of 30 DAS hydroponically grown maize (*Zea mays* cv. Caramelo) plants in response to salinity (non-salinity stress, 0 mM; salinity stress, 60 mM) and biostimulant applications (SA, salicylic acid (25 µM); WB, willow bark extract (0.1% or 0.2% *w/v*); WL, willow leaf extract (0.1% or 0.2% *w/v*)).

Treatments	Salinity (mM)	Fe (mg.kg^−1^)	Zn (mg.kg^−1^)	Mn (mg.kg^−1^)	Cu (mg.kg^−1^)	Mo (mg.kg^−1^)
Control	0	83 ± 6 bc	53 ± 7 b–d	102 ± 7 de	8.5 ± 0.7 a	2.29 ± 0.43 a
	60	130 ± 37 a	90 ± 13 a	157 ± 21 a–c	8.3 ± 1.0 a	0.57 ± 0.19 b
SA (25 µM)	0	73 ± 4 bc	42 ± 2 d	94 ± 7 e	8.5 ± 0.5 a	2.05 ± 0.13 a
	60	120 ± 31 a	68 ± 11 bc	149 ± 23 a–d	9.3 ± 2.2 a	0.49 ± 0.13 b
WB (0.1%)	0	69 ± 15 bc	48 ± 4 cd	124 ± 25 b–e	8.3 ± 0.8 a	2.32 ± 0.26 a
	60	95 ± 9 ab	69 ± 6 b	181 ± 35 a	8.5 ± 1.5 a	0.57 ± 0.13 b
WB (0.2%)	0	58 ± 3 c	40 ± 5 d	108 ± 7 c–e	6.9 ± 0.4 a	2.22 ± 0.24 a
	60	76 ± 11 bc	45 ± 6 d	153 ± 14 a–d	7.1 ± 0.3 a	0.37 ± 0.12 b
WL (0.1%)	0	62 ± 2 bc	51 ± 3 b-d	124 ± 26 b–e	9.4 ± 0.2 a	2.24 ± 0.12 a
	60	70 ± 8 bc	65 ± 15 bc	173 ± 8 ab	9.7 ± 2.5 a	0.52 ± 0.13 b
WL (0.2%)	0	61 ± 7 bc	44 ± 6 d	126 ± 28 b–e	9.4 ± 0.5 a	2.39 ± 0.14 a
	60	66 ± 14 bc	43 ± 5 d	168 ± 25 ab	7.7 ± 1.0 a	0.33 ± 0.14 b

Reported data are means of 4 independent biological replicates. Different letters indicate significant differences between means according to Tukey’s HSD test (*p* < 0.05). For two-way ANOVA results and the HSD scores used to separate the means, see Table 1.

**Table 7:** The mineral element concentration of the willow extracts.

Elements		Extracts	
		WB	WL
Macronutrient Concentrations (%)	Ca	0.001 ± 0.001	0.023 ± 0.002
	K	0.018 ± 0.000	0.057 ± 0.014
	Mg	0.002 ± 0.000	0.006 ± 0.000
	P	0.005 ± 0.000	0.003 ± 0.000
	S	0.010 ± 0.001	0.038 ± 0.003
Micronutrient Concentrations (mg.L^−1^)	B	0.082 ± 0.380	2.360 ± 0.246
	Cu	0.040 ± 0.043	0.083 ± 0.091
	Fe	0.038 ± 0.065	0.030 ± 0.027
	Mn	0.139 ± 0.015	1.326 ± 0.098
	Zn	0.709 ± 0.196	0.000 ± 0.007
	Mo	0.007 ± 0.012	0.002 ± 0.003

## Data Availability

The data presented in this study are available on request from the corresponding author.
